# Ovarian metastases from renal cell carcinoma: A report of two cases

**DOI:** 10.20407/fmj.2024-033

**Published:** 2025-08-06

**Authors:** Junichi Takagi, Ryoko Ichikawa, Kyohei Takada, Akiko Ohwaki, Mayuko Ito, Yutaka Torii, Hiroyuki Nomura, Haruki Nishizawa

**Affiliations:** 1 Department of Obstetrics and Gynecology, Fujita Health University, School of Medicine, Toyoake, Aichi, Japan; 2 Department of Obstetrics and Gynecology, Tokai University School of Medicine, Isehara, Kanagawa, Japan

**Keywords:** Renal cell carcinoma, Ovarian metastasis, Metastatic ovarian tumor

## Abstract

Renal cell carcinoma is a malignant tumor that is prone to distant metastasis, primarily developing in the lungs, bones, lymph nodes, liver, and brain. Conversely, ovarian metastasis is rare, and its clinical characteristics and optimal treatment strategies remain unestablished. We report two cases with renal cell carcinoma and concomitant ovarian metastasis. Case 1 was of a 52-year-old woman with a metastatic tumor in the left ovary detected 9 years after radical nephrectomy for left-sided clear cell renal cell carcinoma. Case 2 was of a 56-year-old woman with a metastatic tumor in the right ovary detected 15 years after radical nephrectomy for right-sided clear cell renal cell carcinoma. In both cases, preoperative imaging revealed a pelvic tumor with a strong contrast effect. Surgery was performed for diagnosis and treatment of suspected ovarian metastasis from the renal cell carcinoma. Rapid intraoperative diagnosis was performed to determine the surgical approach, and the diagnosis of ovarian metastasis from renal cell carcinoma was made. Subsequently, bilateral adnexectomy was performed, and following pathological examination led to the definite diagnosis of ovarian metastasis from clear cell renal cell carcinoma. Long after radical nephrectomy, ovarian metastasis from the renal cell carcinoma can occur. Therefore, continuous follow-up is important. Complete resection of ovarian metastases from renal cell carcinoma may contribute to improved prognosis; however, more case studies are needed to establish standard treatment.

## Introduction

Renal cell carcinoma is a malignant tumor with increased incidence of distant metastasis, occurring in 10%–28% of patients even after radical nephrectomy.^1–4^ Reportedly, the distribution of metastatic sites of renal cell carcinoma is highest in the lungs (45.2%), followed by the bones (29.5%), lymph nodes (21.8%), liver (20.3%), adrenal glands (8.9%), and brain (8.1%).^5^ Conversely, studies have reported that ovarian metastasis is extremely rare, occurring in 0.2% of cases with metastatic renal cell carcinoma.^5^

Differentiating ovarian metastasis from renal cell and primary ovarian carcinoma is important to determine an appropriate treatment strategy and assess prognosis. However, there are limitations to preoperative diagnosis based on imaging procedures. Moreover, to determine the most appropriate surgical procedure, intraoperative findings and rapid intraoperative pathology are indispensable.

In this report, we present the clinical course of two cases with ovarian metastasis originating from renal cell carcinoma. Additionally, we discuss the characteristics, diagnosis, treatment, and challenges of ovarian metastasis originating from renal cell carcinoma.

## Case 1

**Figure 1:**
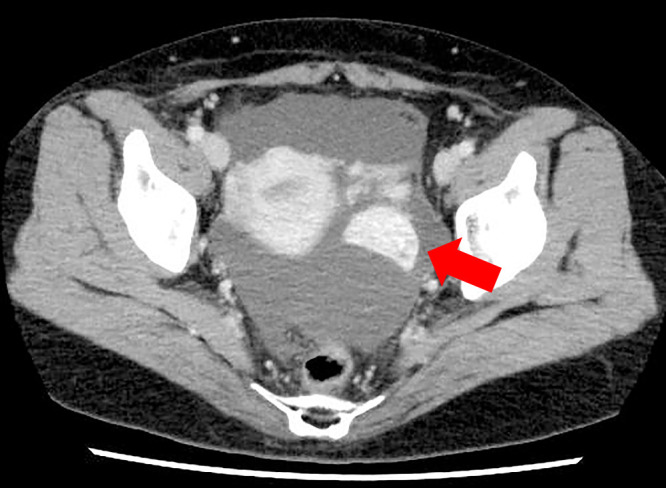
Contrast-enhanced computed tomography image of the pelvic region (Case 1) A solid tumor with strong contrast enhancement was found in the left pelvis.

A 52-year-old woman underwent laparoscopic radical left nephrectomy for a left kidney tumor of 79 mm in size 9 years ago, and the pathological diagnosis was clear cell renal cell carcinoma. Postoperatively, no additional treatment was required and follow-up was performed. Two years ago, she underwent robot-assisted pancreaticoduodenectomy for pancreatic metastasis of renal cell carcinoma. Thereafter, contrast-enhanced computed tomography (CT) revealed a solid tumor with strong contrast effect in the left pelvis (Figure 1), and the patient was referred to our department for close examination and treatment.

**Figure 2:**
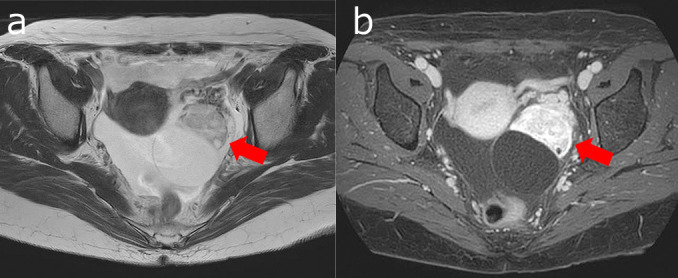
Magnetic Resonance Imaging of the pelvic region (Case 1) A 96-mm sized solid tumor with some cystic components was found in the left pelvis. T2-weighted imaging revealed isointense and hyperintense signal areas with strong contrast enhancement. Although ovarian malignancy was suspected, it was difficult to differentiate primary and metastatic ovarian tumor. a: T2-weighted horizontal section, b: T1 contrast-enhanced horizontal section

**Figure 3:**
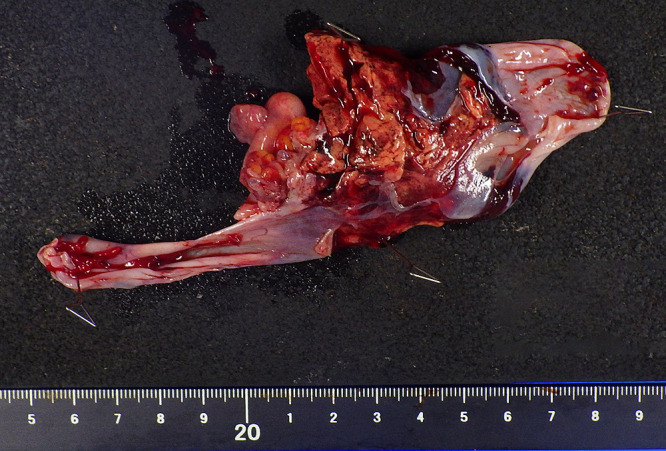
Macroscopic image of the left adnexa (Case 1) The cut surface of the left ovarian tumor was yellowish and hemorrhagic.

**Figure 4:**
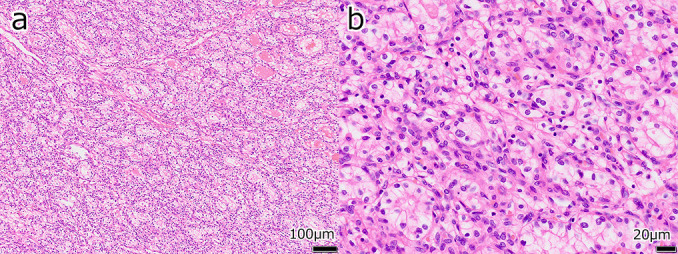
Histopathological findings of the left ovarian tumor (Case 1) Cells with round or partially irregular nuclei, and clear cytoplasm that were proliferating in a solid pattern. a: Hematoxylin and eosin (H&E) stain, low-power; b: H&E stain, high-power enlarged

Pelvic contrast-enhanced magnetic resonance imaging (MRI) revealed ascites in the pelvis and a 96-mm solid tumor with a partially cystic component in the left pelvis. The solid component showed strong contrast effect (Figure 2). Tumor markers were CEA <1.7ng/ml and CA19-9 13.0U/ml, which were within normal range; however, CA125 was elevated at 871.9U/ml (normal range: <35U/ml). Surgical resection was planned for diagnosis and treatment of the suspected left ovarian malignancy. Clear peritoneal dissemination was not present, and abdominal bilateral adnexectomy was performed. The rapid intraoperative pathology results were ovarian metastasis of renal cell carcinoma. The cut surfaces of the left ovarian tumor were yellowish and hemorrhagic (Figure 3). Histopathology revealed cells with round or partially irregular nuclei and clear cytoplasm that were proliferating in a solid pattern (Figure 4). The final diagnosis was ovarian metastasis of clear cell renal cell carcinoma. Post-treatment, the patient has been recurrence-free for 2 years without any adjuvant therapy.

## Case 2

**Figure 5:**
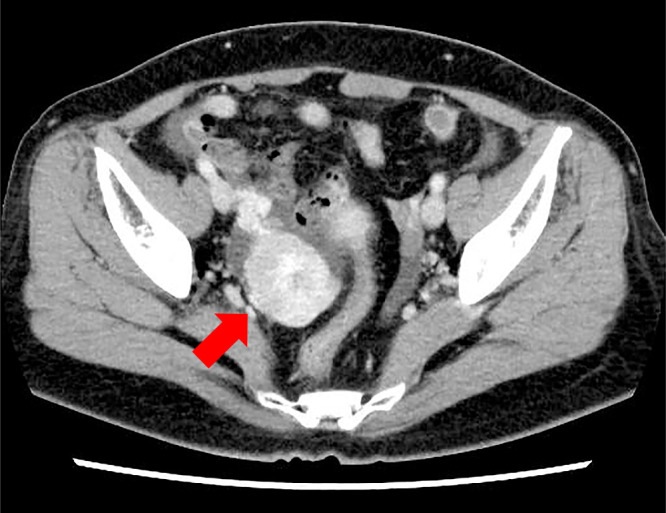
(Case 2) Contrast-enhanced computed tomography scan of the pelvic region (Case 2) A solid tumor of 48 mm in size with abundant blood flow was found in the right pelvis.

A 56-year-old woman with a history of left adnexectomy for ectopic pregnancy underwent radical right nephrectomy for a right renal tumor 15 years ago, which was diagnosed as clear cell renal cell carcinoma. She had undergone right thoracic tumor resection for right lung metastasis and rib metastasis 6 years ago. Thereafter, metastasis was found in the right lung and cervical spine; however, complete remission was achieved after treatment with sunitinib malate. During follow-up, a contrast-enhanced CT scan revealed a 48-mm solid tumor in the right pelvis with abundant blood flow (Figure 5). Consequently, the patient was referred to our department for close examination and treatment.

**Figure 6:**
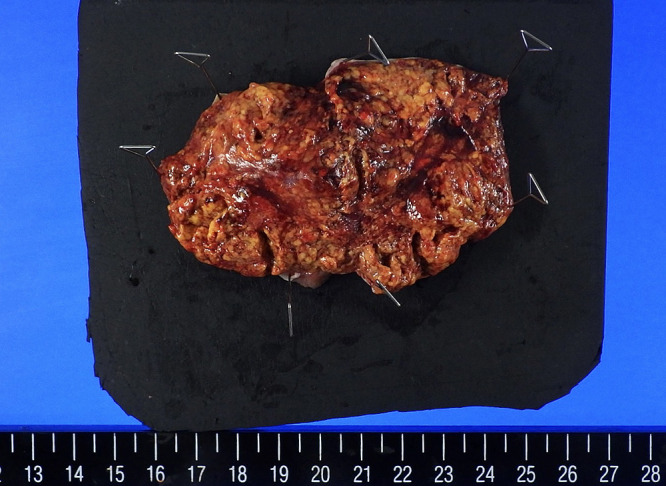
Macroscopic image of the right ovarian tumor (Case 2) The right ovarian tumor was yellowish, spongy and hemorrhagic.

**Figure 7:**
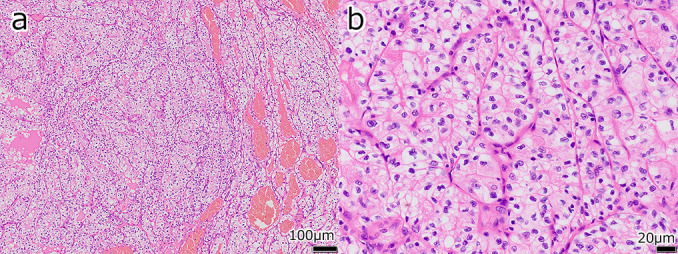
Histopathological findings of the right ovarian tumor (Case 2) Tumor cells with round or partially irregular nuclei and clear to slightly eosinophilic cytoplasm were growing in a solid pattern, forming alveolar and glandular duct structures. The tumor was divided by fibrovascular stroma. a: Hematoxylin and eosin (H&E) stain, weakly enlarged; b: H&E stain, strongly enlarged

Tumor marker CA125 was within normal range at 25.9 U/ml. The patient had an intrauterine contraceptive device that could not be removed, making MRI imaging difficult. Surgical resection was planned for diagnosis and treatment of the suspected primary tumor of the left ovary or ovarian metastasis of renal cell carcinoma. Abdominal right adnexectomy was performed, and the rapid pathological diagnosis was ovarian metastasis of renal cell carcinoma. Moreover, clear peritoneal dissemination was not found. The right ovarian tumor had yellowish and spongy, hemorrhagic cut surfaces (Figure 6). Histopathological findings revealed tumor cells with round or partially irregular nuclei, and clear to slightly eosinophilic cytoplasm that were proliferating in a solid pattern, forming alveolar- and glandular duct–like structures. The tumor was divided by fibrovascular stroma (Figure 7). The final diagnosis was ovarian metastasis of clear cell renal cell carcinoma. Post-treatment, the patient has been recurrence-free for 2 years without any adjuvant therapy.

## Discussion

Although renal cell carcinoma is a tumor that is prone to distant metastasis, ovarian metastasis is rare. Even in a large-scale study by Saito H on 1451 autopsy cases of renal cell carcinoma that included 324 women, there were no cases of ovarian metastasis.^6^ The pathological and physiological factors underlying the rarity of ovarian metastasis from renal cell carcinoma are discussed. Renal cell carcinoma commonly manifests in patients in their 60s–70s, when the ovaries exhibit age-related fibrotic and atrophic changes. Postmenopause, the ovaries lose weight and blood flow to the ovaries is markedly reduced. Thus, it is assumed that these menopausal changes limit tumor emboli from reaching the ovaries, which is the primary mechanism of hematogenous ovarian metastasis, resulting in decreased incidence of ovarian metastasis.^7^ Clinically, this hypothesis is supported by the observation that the median age at Kruckenberg’s tumor diagnosis, accounting for 30%–40% of metastatic ovarian tumors, is 48 years (range: 27–40). Moreover, metastatic ovarian tumors are more common reported in age groups where ovarian blood flow is preserved.^8,9^ Table 1 presents the findings of 41 cases with ovarian metastases from renal cell carcinoma, including a review of the literature and our own cases. The median age was 52 years (range: 17–82 years), which is younger than the typical age of onset for renal cell carcinoma (i.e., 60s–70s), suggesting that the frequency of ovarian metastases increases at an age before the presentation of age-related changes in the physiological state of the ovaries. Generally, metastatic ovarian tumors tend to be bilateral, and a study on mucinous ovarian carcinoma reported that 87.4% of patients with bilateral ovarian tumors had metastatic ovarian tumors.^10^ Conversely, in the 41 cases analyzed (Table 1), the distribution of metastatic lesions was predominantly unilateral (11 bilateral and 29 unilateral ovaries), including 13 ipsilateral and 14 contralateral ovaries. This suggests that metastatic ovarian tumors may have different characteristics depending on the primary site and histology. Furthermore, among the patients with unilateral ovarian metastasis, 17 had left-sided ovarian metastasis and 12 had right-sided ovarian metastasis, indicating a left-sided predominance. This left-sided predominance suggests the existence of a retrograde hematogenous metastatic route via the left ovarian vein that flows directly into the left renal vein. However, the observed contralateral and bilateral ovarian metastasis suggesting that the presence of vascular plexus between the bilateral ovaries contribute to the metastasis.^7^

The time from renal cell carcinoma diagnosis to ovarian metastasis varied from 3 months to 21 years. Levy et al. reported that patients developed distant metastasis after surgical resection of renal cell carcinoma within a median postoperative period of 23 months.^2^ Kim et al. reported that after an initial 5-year recurrence-free period following surgical resection of renal cell carcinoma, 15% of patients developed distant metastasis during the following 10 years.^11^ Although the appropriate duration of postoperative follow-up for renal cell carcinoma is not clear, cases 1 and 2 developed ovarian metastases 5 years after the surgery for renal cell carcinoma, suggesting the importance of long-term follow-up. Therefore, in premenopausal patients with renal cell carcinoma, regular imaging examinations of the pelvic region should be performed to screen for ovarian metastasis.

Kajiyama et al. reported that colorectal (43%) and gastric cancers (29%) are the most common primary sites of metastatic ovarian cancer, followed by appendix (8%), breast (6%), and pancreatic cancers (4%).^12^ Although metastatic ovarian cancer is generally associated with a poor prognosis, standard treatment strategies have not yet been established. In a study on ovarian metastases from colorectal cancer, the median survival rate was 48 months in patients who had en bloc tumor resection, compared with 8 months in patients with diffuse disease extending beyond the pelvis. These results suggest that en bloc resection of lesions contributes to improved survival.^13^ Additionally, the lower response rate to chemotherapy for ovarian metastases (5%) than that for extra-ovarian disease (42%–58%) in patients with primary colorectal cancer suggests that surgical resection for ovarian metastases is beneficial as a palliative treatment.^14^ Currently, no clear evidence has been established with regard to the efficacy of surgical intervention for ovarian metastases from renal cell carcinoma. However, there are reports suggesting the usefulness of en bloc resection for metastatic lesions of renal cell carcinoma. Alt et al. reported that en bloc resection of distant metastases from renal cell carcinoma significantly prolonged median survival compared with the nonresection group (4.8 years vs. 1.3 years; p<0.001). Furthermore, the 5-year survival rate was higher in the en bloc resection group than in the nonresection group for single and multiple lung metastases.^15^ Daliani et al. found a marked difference in median survival between the en bloc and nonen bloc resection groups of metastatic lesions in renal cell carcinoma patients (5.6 years vs. 1.4 years; p<0.001), suggesting that en bloc resection of metastatic lesions may contribute to improved prognosis.^16^

Differentiating ovarian metastasis of renal cell carcinoma from primary ovarian carcinoma is important to determine the appropriate treatment strategy and in assess its prognosis. In fact, there have been more reports of patients who underwent surgery for suspected primary ovarian cancer at the initial stage than who were diagnosed with ovarian metastasis from renal cell carcinoma through postoperative close examination.^17–19^ In general, it is often difficult to make a tissue-specific diagnosis of ovarian tumors using imaging studies. Therefore, in addition to preoperative evaluation and intraoperative intraperitoneal findings, a rapid intraoperative pathologic diagnosis is useful to determine the optimal surgical approach. According to a study by Ilvan et al., the accuracy of rapid pathological diagnosis of ovarian tumors (n=617), benign or malignant, was 97%. The sensitivity for benign, borderline malignant, and malignant tumors was reported to be 100%, 87%, and 87%, respectively. Moreover, 20 of the 120 malignant tumors were metastatic, and the primary sites were the colon in eight cases, stomach in seven cases, and breast in five cases, and 19 out of 20 (95%) metastatic tumors were correctly diagnosed as metastatic using rapid pathology, and only one case of fibroma using rapid pathology was inconsistent with the final diagnosis.^20^ In a study by Yoshida et al., 57 of 69 (82.6%) metastatic tumors were correctly diagnosed as metastatic using rapid pathology. The primary tumor in the cases that were inconsistent with the final diagnosis was of the large intestine (five cases), the appendix (five cases), and the uterine body (two cases).^21^ In both of our cases, metastatic ovarian cancer was considered based on their history of renal cell carcinoma and imaging findings, and appropriate procedures could be performed based on the intraoperative rapid pathology diagnosis. In reports of ovarian metastases from renal cell carcinoma, CA125 levels were often within the normal range, making it particularly important to distinguish them from primary ovarian carcinoma. It has been reported that elevated CA125 is correlated with ascites as well as ovarian and peritoneal cancer, and in case 1, the abnormally high value was thought to be associated with ascites accumulation.^22^

Reportedly, the most common type of renal cell carcinoma is clear cell renal cell carcinoma, accounting for approximately 75% of all renal cell carcinomas.^23^ Conversely, clear cell ovarian carcinoma is relatively rare among epithelial ovarian carcinomas in the western countries, accounting for approximately 6% of all epithelial ovarian cancers. Whereas, in Japan, it tends to occur at a high frequency of approximately 25%.^10^ There are important histopathological distinctions between these two types of cancer. Macroscopically, ovarian clear cell carcinoma typically forms smooth-surfaced cyst with a yellowish-white protrusion into the lumen. In contrast, clear cell renal cell carcinoma is characterized by a yellowish cut surface, often with hemorrhage within the tumor. Histopathologically, clear cell carcinoma of the ovary presents with a variety of histological structures such as papillary, tubular cystic, or solid structures, and remarkable nuclear atypia. Characteristically, hobnail cells with nuclei protruding toward the glandular lumen may be observed. Conversely, in clear cell renal cell carcinoma, tumor cells with a clear cytoplasm proliferate in alveolar, solid, or tubular structures, and a characteristic network of capillaries surrounding the tumor cells is observed. In some cases, it is difficult to distinguish between renal cell and ovarian clear cell carcinoma based on histopathology alone, and immunohistochemical staining may be useful in such cases. According to a study by Nolan et al., there are immunohistological differences between renal cell and ovarian clear cell carcinoma. Specifically, vimentin (8/12 cases), 34βE12 (1/12 cases), CA125 (0/12 cases), ER (1/12 cases), and PgR (1/12 cases) are positive in renal cell carcinoma, whereas vimentin (1/10 cases), 34βE12 (10/10 cases), CA125 (8/10 cases), ER (7/10 cases), and PgR (6/10 cases) tend to be positive in ovarian clear cell carcinoma.^24^ In our cases, the histopathological features characteristic of clear cell renal cell carcinoma were observed, making the diagnosis possible. However, when a differential diagnosis is difficult to make, additional immunohistochemical staining may be helpful.

Although ovarian metastasis of renal cell carcinoma is rare, long-term follow-up is needed considering its possibility, especially in premenopausal patients diagnosed with renal cell carcinoma. Based on preoperative imaging findings, intraoperative findings, and intraoperative rapid pathology results, it is essential to carefully differentiate between ovarian metastasis of renal cell carcinoma and primary ovarian cancer, and to determine the appropriate surgical technique after considering the possibility of both types of cancers. Although there is no standard treatment for metastatic ovarian tumors, adnexectomy for en bloc resection of metastases may be an effective treatment option for patients with ovarian metastases originating from renal cell carcinoma. Therefore, more case studies are warranted to establish the efficacy of adnexectomy for renal cancer.

## Figures and Tables

**Table 1:** The summary of the cases

References	Age (years)	Laterality of RCC	Laterality of Ovarian metastasis	First detected site	Time to metastases
Vorder 1957	64	Right	Bilateral	Kidney	11 y
Stefani 1981	68	Right	Left	Kidney	3 mo
Young 1992	48	Right	Left	Ovary	8 mo
	62	Left	Right	Kidney	1 y
	48	Left	Left	Synchronous	
Liu 1992	28	Right	Left	Kidney	7 mo
Spencer 1993	40	Left	Bilateral	Ovary	7 mo
Adachi 1994	46	Left	Bilateral	Kidney	3 y
Fields 1996	54	Right	Left	Kidney	3 y
Vara 1998	66	Right	Bilateral	Kidney	14 y
Hammock 2003	48	Left	Right	Synchronous	
Insabato 2003	50	Right	Right	Kidney	1 y
	49	Right	NA	Kidney	14 mo
	17	Left	Left	Kidney	2 y
Valappil 2004	61	Left	Bilateral	Kidney	7 y
Kato 2006	52	Left	Right	Synchronous	
Stolnicu 2007	73	NA	Left	Kidney	NA
Toquero 2009	54	Left	Left	Synchronous	
Albrizio 2009	56	Right	Bilateral	Kidney	10 y
Decoene 2011	47	Right	Left	Kidney	5 y
Bauerova 2014	61	Right	Bilateral	Kidney	21 y
Bohara 2015	48	Right	Right	Kidney	3 y
Kostrzewa 2015	51	Left	Right	Kidney	4 y
Liang 2016	60	Right	Right	Synchronous	
	NA	NA	Bilateral	Synchronous	
	48	Left	Right	Kidney	14 mo
	37	Left	Bilateral	Kidney	8 mo
	45	Right	Left	Kidney	30 mo
	43	Right	Right	Kidney	20 mo
	52	Left	Right	Kidney	10 mo
	52	Right	Left	Ovary	NA
Uruc 2017	48	Right	Left	Kidney	22 mo
Bhaskar 2017	45	Left	Left	Synchronous	
Porfyris 2018	82	Right	Left	Synchronous	
Karaosmanoglu 2019	52	Left	Left	Kidney	4 y
Takayanagi 2019	66	Right	Right	Kidney	4 y
Fujii 2021	58	Right	Bilateral	Kidney	8 y
Snyder 2021	48	Left	Bilateral	Synchronous	
Younes 2023	56	Left	Left	Kidney	15 y
Present case 2022	52	Left	Left	Kidney	9 y
	56	Right	Right	Kidney	15 y

Abbreviation: NA; not available, RCC; renal cell carcinoma, y; year, mo; month

## Supplementary Material

PDF-Japanese
