# Supplementary material for: Ovarian metastases from renal cell carcinoma: A report of two cases
Source: Fujita Med J. 2025 Aug 6;11(4):193–8. doi: 10.20407/fmj.2024-033 (PMC12576397; doi:10.20407/fmj.2024-033)
Supplement: Supplementary file 1 — PDF-Japanese [file fmj-11-193-s001.pdf]

Case report

Title：Ovarian Metastases from Renal Cell Carcinoma: A Report of Two Cases

Running Head：Ovarian Metastases from Renal Cell Carcinoma: A Report of Two Cases

Junichi Takagi, MD[1], Ryoko Ichikawa, MD[1], Kyohei Takada, MD[1], Akiko Ohwaki, MD, PhD[1], Mayuko Ito, MD, PhD[1], Yutaka Torii, MD[1], Hiroyuki Nomura, MD, PhD[2], Haruki Nishizawa, MD, PhD[1]

[1]Department of Obstetrics and Gynecology , Fujita Health University, School of Medicine, Toyoake, Aichi, Japan

[2]Department of Obstetrics and Gynecology , Tokai University School of Medicine, Isehara, Kanagawa, Japan

Corresponding author: Junichi Takagi, MD

Department of Obstetrics and Gynecology, Fujita Health University, School of Medicine, 1-98, Dengakugakubo, Kutsukake-cho, Toyoake, Aichi 470-1192, Japan

Tel: 0562-93-9294

E-mail: junichi.takagi@fujita-hu.ac.jp

要旨

腎細胞癌は遠隔転移を来たしやすい悪性腫瘍として知られており，主に肺，骨，リンパ節，肝臓，脳への転移が報告されている．一方，卵巣転移は稀少であり，その臨床的特徴や最適な治療戦略は十分に確立されていない．今回腎細胞癌の卵巣転移を呈した 2 症例を経験したので報告する．症例 1 は 52 歳女性で，左淡明細胞型腎細胞癌に対して根治的腎摘除術後 9 年で左卵巣に転移性腫瘍を認めた．症例 2 は 56 歳女性で，右淡明細胞型腎細胞癌に対して根治的腎摘除術後 15 年で右卵巣に転移性腫瘍を認めた．両症例とも術前の画像検査で強い造影効果を伴う骨盤内腫瘍を認め，腎細胞癌の卵巣転移を疑い，診断および治療目的に手術を行った．術式の決定のために術中迅速診断を行い，腎細胞癌の卵巣転移の診断であった．両側付属器摘出術を施行し病理検査により淡明細胞型腎細胞癌の卵巣転移の確定診断に至った．腎細胞癌の卵巣転移は根治的腎摘除術の長期経過後にも発生し得るため，継続的な経過観察が重要である．腎細胞癌の卵巣転移巣の完全切除が予後改善に寄与する可能性があるが，標準治療の確立には更なる症例の蓄積が必要である．

キーワード：腎細胞癌，卵巣転移，転移性卵巣腫瘍

緒言

腎細胞癌は高頻度に遠隔転移を呈する悪性腫瘍であり，根治的腎摘除術後においても，10%から 28%の患者に遠隔転移が認められる．[1-4] 腎細胞癌の転移部位の頻度分布は肺(45.2%)，骨(29.5%)，リンパ節(21.8%)，肝臓(20.3%)，副腎(8.9%)，脳(8.1%)の順に高いことが報告されている．[5] 一方，卵巣転移は極めて稀であり，転移を有する腎細胞癌症例の 0.2%のみに認められたと報告されている．[5]

腎細胞癌の卵巣転移と原発性卵巣癌との鑑別は，適切な治療方針の決定と予後の評価において重要な意義を持つが，その一方で，画像検査による術前診断には限界があり，最適な術式を決定するためには，術中所見や術中迅速病理診断が不可欠となる．

本報告では，腎細胞癌の卵巣転移を認めた 2 症例について，その臨床経過について詳述し，腎細胞癌の卵巣転移の特徴，診断，治療，およびその課題について考察を加えて報告する．

症例 1

52 歳女性．9 年前に 79mm の左腎腫瘍に対して腹腔鏡下根治的左腎摘除術を施行し，病理診断は淡明細胞型腎細胞癌であった．術後追加治療なく経過観察となり，2 年前に腎細胞癌膵臓転移に対してロボット支援下膵頭十二指腸切除術を施行した．その後，造影 CT 検査で左骨盤内に強い造影効果を伴う充実性腫瘍を認め（Figure1），精査加療目的に当科へ紹介となった．

骨盤部造影 MRI 検査では骨盤内に腹水を認め，左骨盤内に 96mm 大の一部嚢胞成分を伴う充実性腫瘍を認め，充実成分は強い造影効果を示した（Figure2）．腫瘍マーカーは CEA <1.7ng/ml，CA19-9 13.0U/ml と正常範囲内であったが，CA125 は 871.9U/ml と上昇していた（正常範囲：35U/ml 未満）．左卵巣悪性腫瘍を疑い，診断および治療目的に外科的切除を計画した．腹腔内に明らかな播種巣は認めず，腹式両側付属器摘出術を施行し，迅速病理診断は腎細胞癌の卵巣転移であった．左卵巣腫瘍の割面は黄色調で出血を伴っていた（Figure3）．病理組織所見では，類円形あるいは一部不整形の核を持ち，淡明な胞体を有する細胞が充実性に増殖しており（Figure4），最終診断は淡明細胞型腎細胞癌の卵巣転移とした．術後補助療法なく，2 年間無再発で経過している．

症例 2

56 歳女性．異所性妊娠に対する左付属器摘出術の既往あり．15 年前に右腎腫瘍に対して根治的右腎摘除術を施行し淡明細胞型腎細胞癌の診断となった．6 年前に右肺転移，肋

骨転移に対して右胸部腫瘍切除術を施行した．その後，右肺，頸椎に転移を認めるも，スニチニブリンゴ酸塩で完全寛解を得た．経過観察中，造影 CT 検査で右骨盤内に 48mm 大の血流豊富な充実性腫瘍を認め（Figure5），精査加療目的に当科へ紹介となった．

　腫瘍マーカーCA125 は 25.9U/ml と正常範囲内であった．除去不能の子宮内避妊器具が留置されており MRI 撮像は困難であった．左卵巣原発腫瘍もしくは腎細胞癌の卵巣転移を疑い，診断および治療目的に外科的切除を計画した．腹式右付属器摘出術を施行し，迅速病理診断は腎細胞癌の卵巣転移であった．腹腔内に明らかな播種巣は認めなかった．右卵巣腫瘍の割面は黄色調で出血を伴うスポンジ状であった（Figure6）．病理組織所見では，類円形あるいは一部不整形の核を持ち，淡明から淡好酸性の胞体を有する腫瘍細胞が胞巣構造や腺管構造を形成しながら充実性に増殖していた．また，腫瘍内は線維性血管間質によって区画されていた（Figure7）．最終診断は淡明細胞型腎細胞癌の卵巣転移とした．術後補助療法なく，2 年間無再発で経過している．

考察

腎細胞癌は遠隔転移を来たしやすい腫瘍であるが，卵巣への転移は稀である．Saitoh らによる 1451 例（女性 324 例を含む）の腎細胞癌剖検例の大規模研究においても卵巣転移例はなかった．[6] 腎細胞癌の卵巣転移が稀少である要因を，病理学的および生理学的に考察する．腎細胞癌の好発年齢である 60 歳から 70 歳代において，卵巣は加齢に伴う線維化および萎縮性変化を呈する．さらに，閉経後には卵巣の重量減少が生じ，卵巣への血流量が顕著に低下する．これらの閉経に伴う変化により，血行性卵巣転移の主要な機序である腫瘍塞栓の卵巣への到達が制限され，結果として卵巣転移の発生頻度が低下すると推測される．[7] この仮説を支持する臨床的観察として，転移性卵巣腫瘍の 30～40%を占めるクルッケンベルグ腫瘍の診断時年齢中央値は 48 歳（範囲：27－65 歳）であり，卵巣血流が保たれている年齢では転移性卵巣腫瘍が多くみられることが報告されている．[8,9] 文献レビューおよび自験例を含む 41 例の腎細胞癌卵巣転移症例の解析結果を Table1 に示す．年齢分布は中央値 52 歳（範囲：17 歳-82 歳）であり，腎細胞癌の一般的な好発年齢（60 歳代および 70 歳代）より若年であったことから，加齢に伴う卵巣の生理学的状態変化が出現する以前の年齢では卵巣転移の頻度が上昇する可能性が示唆される．一般的に転移性卵巣腫瘍は両側性を呈することが多いとされており，Shimons らは粘液性卵巣癌について検討を行い，両側性卵巣腫瘍を呈した場合に 87.4%が転移性卵巣腫瘍であったと報告している．[10] 一方で，Table1 に示した 41 例の解析結果では，転移巣の分布は，両側卵巣 11 例，片側

卵巣 29 例（うち同側卵巣 13 例，対側卵巣 14 例）と片側性が優位であった．このことは，転移性卵巣腫瘍が原発巣や組織型によって異なる特徴を呈する可能性を窺わせる．さらに，片側卵巣転移例のうち左卵巣転移が 17 例，右卵巣転移が 12 例と左側優位の傾向が認められた．この左側優位性は，左腎静脈に直接流入する左卵巣静脈を介した逆行性の血行性転移経路の存在を示唆する．一方，対側卵巣への転移や両側卵巣転移も認められ，両側卵巣間の血管叢の存在が寄与している可能性がある．[7]

腎細胞癌の診断から卵巣転移を認めるまでの期間は 3 か月から 21 年と多様であった．Levy らの報告によると，腎細胞癌の外科的切除後に遠隔転移を認めた患者の術後期間の中央値は 23 か月であった．[2]Kim らは腎細胞癌の外科的切除後の最初の 5 年間の無病期間の後，その後の 10 年間に 15%の患者に遠隔転移を認めたと報告している．[11]腎細胞癌に対する術後フォローアップの適切な期間は明確ではないが，症例 1 および 2 はともに腎細胞癌の手術から 5 年以降に卵巣転移を認めており，長期間の経過観察の重要性が示唆される．中でも，閉経前に発症した腎細胞癌患者においては，卵巣転移を考慮し，定期的な骨盤部も含めた画像検査の実施が必要と考えられた．

転移性卵巣癌の原発巣としては，Kajiyama らの報告によると大腸癌（43%），胃癌（29%）が高頻度であり，続いて虫垂癌（8%），乳癌（6%），膵臓癌（4%）が挙げられる．[12] 転移性卵巣癌は一般的に予後不良とされているが標準的な治療戦略は未だ確立されていない．大腸癌由来の卵巣転移に関する研究では，腫瘍の完全切除が可能であった患者の

生存期間中央値が 48 ヶ月であったのに対し，骨盤外に及ぶびまん性病変を有する患者では 8 ヶ月であった．この結果は，病変の完全切除が生存期間の改善に寄与することを示唆している．[13] また，原発性大腸癌患者の卵巣外病変に対する化学療法の奏効率（42-58%）と比較し，卵巣転移に対する奏効率は低い（5%）ことから，卵巣転移の外科的切除が緩和治療として有用であることが考えられる．[14] 腎細胞癌の卵巣転移に対する外科的介入の有効性については，現時点で明確なエビデンスは確立されていない．しかしながら，腎細胞癌の転移巣に対する完全切除の有用性を示唆する報告が存在する．Alt らによると，腎細胞癌の遠隔転移に対する完全切除術は，非切除群と比較して生存期間の中央値を有意に延長させた（4.8 年 vs1.3 年；p<0.001）．さらに，5 年生存率においても，肺単独転移例および多発性転移例の両方で，完全切除群が非切除群を上回る結果が示されている．[15]Daliani らの報告でも，腎細胞がん患者における転移巣の完全切除群と不完全切除群の生存期間中央値に顕著な差が認められ（5.6 年 vs 1.4 年；p<0.001），転移巣の完全切除が予後改善への寄与が示唆されている．[16]

腎細胞癌の卵巣転移と原発性卵巣癌との鑑別は，適切な治療方針の決定と予後の評価において重要な意義を持つ．実際に，初期段階で原発性卵巣癌を疑い手術を実施したものの，術後の精査により腎細胞癌の卵巣転移と診断される症例が報告されている．[17-19] 一般的に卵巣腫瘍は画像検査による組織特異的な診断が困難なことが多い．したがって，最適な術式を決定するためには，術前評価と術中の腹腔内所見に加えて，術中迅速病理診断が有用

である． Ilvan らの研究によると，卵巣腫瘍全体（n=617）における迅速病理診断の良悪性の正診率は97%であった．良性腫瘍，境界悪性腫瘍，悪性腫瘍の感度はそれぞれ100%，87%，87%と報告されている．悪性腫瘍 120 例中 20 例が転移性であり，原発巣の内訳は大腸 8 例，胃 7 例，乳房 5 例であった．転移性腫瘍 20 例中 19 例（95%）が迅速病理診断で転移性と正しく診断され，迅速病理診断では線維腫と診断された 1 例のみが最終診断で不一致であった．[20]Yoshida らの研究では，転移性腫瘍 69 例中 57 例（82.6%）が迅速病理診断で転移性腫瘍と正しく診断された．最終診断と不一致であった症例の原発巣は大腸 5 例，虫垂 5 例，子宮体部 2 例であった．[21]本症例においては，2 例とも腎細胞癌の既往と画像所見から転移性卵巣癌を考慮し，術中迅速病理診断に基づいて適切な術式が施行可能であった． また，症例 1 では CA125 の高値を認めた．腎細胞癌の卵巣転移の報告では CA125 は正常範囲であることが多く，原発性卵巣癌との鑑別が特に重要であった．CA125 の上昇は卵巣癌や腹膜癌の他に，腹水の存在に相関しているという報告もあり，症例 1 では腹水貯留に伴う異常高値と考えられた．[22]

腎細胞癌の中で最も頻度が高いのは淡明細胞型腎細胞癌であり，腎細胞癌の約 75%を占めると報告されている．[23]一方，卵巣明細胞癌は欧米では上皮性卵巣癌の中では比較的稀で，上皮性卵巣癌の約 6%とされている．ただし，日本においては約 25%と高頻度で発生する傾向が認められる．[10]これら二つの癌は，病理組織学的に重要な鑑別点がある．肉眼的特徴として，卵巣明細胞癌は典型的に表面平滑な嚢胞を形成し，その内腔に黄白色の充

実性隆起を呈する．対照的に，淡明細胞型腎細胞癌は割面が黄色を呈し，しばしば腫瘍内部に出血を伴うことが特徴的である．病理組織所見では，卵巣明細胞癌は乳頭状，管状嚢胞状，あるいは充実性など多様な組織構築を示し，核の異型性が顕著である．特徴的な所見として，核が腺腔方向に突出する hobnail 細胞が観察されることがある．一方，淡明細胞型腎細胞癌では，淡明な細胞質を有する腫瘍細胞が胞巣状，充実性，管状構造を呈して増殖し，腫瘍細胞を取り囲む特徴的な毛細血管網が認められる．病理組織像のみでは両者の鑑別が困難な症例も存在し，そのような場合には免疫組織化学染色が有用であると考えられている．Nolan らの研究によれば，腎細胞癌と卵巣明細胞癌の免疫組織学的には差異が認められる．具体的には，腎細胞癌では vimentin (8/12 例)，34βE12 (1/12 例)，CA125 (0/12 例)，ER (1/12 例)，PgR (1/12 例)が陽性を示すのに対し，卵巣明細胞癌では vimentin (1/10 例)，34βE12 (10/10 例)，CA125 (8/10 例)，ER (7/10 例)，PgR (6/10 例)が陽性を示す傾向がある．[24] 本症例においては，淡明細胞型腎細胞癌に特徴的な病理組織像が観察されたため診断が可能であった．しかし，鑑別診断が困難な症例に遭遇した場合には，免疫組織化学染色による追加検査が診断の一助となると考えられる．

　腎細胞癌の卵巣転移は稀であるが，特に閉経前に腎細胞癌と診断された患者においては，その可能性を考慮し，長期的な経過観察が必要である．術前の画像診断所見を基に，術中所見および術中迅速病理診断結果から腎細胞癌の卵巣転移と原発性卵巣癌を慎重に鑑別し，両疾患の可能性を考慮した上で適切な術式を決定することが肝要である．転移性卵巣腫瘍

に対する標準治療法は未だ確立されていないが，腎細胞癌の卵巣転移症例においては，転移巣の完全切除を目的とした付属器摘出術が有効な治療選択肢となり得る．腎癌に対する付属器摘出術の有効性を確立するためには，更なる症例の蓄積が必要である．

利益相反

著者らに開示すべき利益相反はない．

引用文献

1. Kattan MW, Reuter V, Motzer RJ, Katz J, Russo P. A postoperative prognostic nomogram for renal cell carcinoma. J Urol 2001; 166: 63-7.
2. Levy DA, Slaton JW, Swanson DA, Dinney CP. Stage specific guidelines for surveillance after radical nephrectomy for local renal cell carcinoma. J Urol 1998; 159: 1163-7.
3. Sorbellini M, Kattan MW, Snyder ME, Reuter V, Motzer R, Goetzl M, McKiernan J, Russo P. A postoperative prognostic nomogram predicting recurrence for patients with conventional clear cell renal cell carcinoma. J Urol 2005 ; 173: 48-51.
4. Zisman A, Pantuck AJ, Wieder J, Chao DH, Dorey F, Said JW, deKernion JB, Figlin RA, Belldegrun AS. Risk group assessment and clinical outcome algorithm to predict the natural history of patients with surgically resected renal cell carcinoma. J Clin Oncol 2002; 20: 4559-66.
5. Bianchi M, Sun M, Jeldres C, Shariat SF, Trinh QD, Briganti A, Tian Z, Schmitges J, Graefen M, Perrotte P, Menon M, Montorsi F, Karakiewicz PI. Distribution of metastatic sites in renal cell carcinoma: a population-based analysis. Ann Oncol 2012: 973-80.

6. Saitoh H. Distant Metastasis of Renal adenocarcinoma. Cancer 1981; 48: 1487-91.
7. Takayanagi A, Kato F, Nozaki A, Matsumoto R, Osawa T, Kuwahara K, Matsuno Y, Asano H, Kato T, Watari H, Abe T, Shinohara N, Kudo K. Imaging findings of ovarian metastasis of primary renal cell carcinoma: A case report and literature review. Radiol Case Rep 2022; 17: 2320-27.
8. Wu F, Zhao X, Mi B, Feng LU, Yuan NA, Lei F, Li M, Zhao X. Clinical characteristics and prognostic analysis of Krukenberg tumor. Mol Clin Oncol 2015; 3: 1323-8.
9. Zulfiqar M, Koen J, Nougaret S, Bolan C, VanBuren W, McGettigan M, Menias C. Krukenberg Tumors: Update on Imaging and Clinical Features. AJR Am J Roentgenol 2020; 215: 1020-9.
10. Simons M, Bolhuis T, De Haan AF, Bruggink AH, Bulten J, Massuger LF, Nagtegaal ID. A novel algorithm for better distinction of primary mucinous ovarian carcinomas and mucinous carcinomas metastatic to the ovary. Virchows Arch 2019; 474: 289-96.
11. Kim SP, Weight CJ, Leibovich BC, Thompson RH, Costello BA, Cheville JC, Lohse CM, Boorjian SA. Outcomes and clinicopathologic variables associated

with late recurrence after nephrectomy for localized renal cell carcinoma. Urology 2011; 78: 1101-6.

12. Kajiyama H, Suzuki S, Utsumi F, Nishino K, Niimi K, Mizuno M, Yoshikawa N, Kawai M, Oguchi H, Mizuno K, Yamamuro O, Shibata K, Nagasaka T, Kikkawa F. Epidemiological overview of metastatic ovarian carcinoma: long-term experience of TOTSG database. Nagoya J Med Sci 2019; 81: 193-8.
13. Morrow M, Enker WE. Late ovarian metastases in carcinoma of the colon and rectum. Arch Surg 1984; 119: 1385-8.
14. Kim DD, Park IJ, Kim HC, Yu CS, Kim JC. Ovarian metastases from colorectal cancer: a clinicopathological analysis of 103 patients. Colorectal Dis 2009; 11: 32-8.
15. Alt AL, Boorjian SA, Lohse CM, Costello BA, Leibovich BC, Blute ML. Survival after complete surgical resection of multiple metastases from renal cell carcinoma. Cancer 2011; 117: 2873-82.
16. Daliani DD, Tannir NM, Papandreou CN, Wang X, Swisher S, Wood CG, Swanson DA, Logothetis CJ, Jonasch E. Prospective assessment of systemic therapy followed by surgical removal of metastases in selected patients with renal cell carcinoma. BJU Int 2009; 104: 456-60.

17. Young RH, Hart WR. Renal cell carcinoma metastatic to the ovary: a report of three cases emphasizing possible confusion with ovarian clear cell adenocarcinoma. Int J Gynecol Pathol 1992; 11: 96–104.

18. Spencer JR, Eriksen B, Garnett JE. Metastatic renal tumor presenting as ovarian clear cell carcinoma. Urology 1993; 41: 582–4.

19. Hammock L, Ghorab Z, Gomez-Fernandez CR. Metastatic renal cell carcinoma to the ovary: a case report and discussion of differential diagnoses. Arch Pathol Lab Med 2003; 127: e123–6.

20. Ilvan S, Ramazanoglu R, Ulker Akyildiz E, Calay Z, Bese T, Oruc N. The accuracy of frozen section (intraoperative consultation) in the diagnosis of ovarian masses. Gynecol Oncol 2005; 97: 395-9.

21. Yoshida H, Tanaka H, Tsukada T, Abeto N, Kobayashi-Kato M, Tanase Y, Uno M, Ishikawa M, Kato T. Diagnostic Discordance in Intraoperative Frozen Section Diagnosis of Ovarian Tumors: A Literature Review and Analysis of 871 Cases Treated at a Japanese Cancer Center. Int J Surg Pathol 2021; 29: 30-8.

22. Emoto S, Ishigami H, Yamashita H, Yamaguchi H, Kaisaki S, Kitayama J. Clinical significance of CA125 and CA72-4 in gastric cancer with peritoneal dissemination. Gastric Cancer 2012; 15: 154-61.

23. Angulo JC, Manini C, López JI, Pueyo A, Colás B, Ropero S. The Role of Epigenetics in the Progression of Clear Cell Renal Cell Carcinoma and the Basis for Future Epigenetic Treatments. Cancers (Basel) 2021; 13: 2071.
24. Nolan LP, Heatley MK. The value of immunocytochemistry in distinguishing between clear cell carcinoma of the kidney and ovary. Int J Gynecol Pathol 2001; 20: 155-9.

図表のタイトル，説明文

Figure1　（症例 1）骨盤部造影 CT 画像

左骨盤内に強い造影効果を伴う充実性腫瘍を認めた．

Figure2　（症例 1）骨盤部 MRI 画像

左骨盤内に 96mm 大の一部嚢胞成分を含む充実性腫瘍を認めた．充実性部分はＴ２強調画像で等信号から高信号を示し，強い造影効果を認めた．卵巣悪性腫瘍を疑うものの卵巣原発腫瘍と転移性卵巣腫瘍との鑑別は困難であった．a：T2 強調像水平断，b：T1 造影後水平断

Figure3　（症例 1）左付属器肉眼写真

左卵巣腫瘍の割面は黄色調で出血を伴っていた．

Figure4　（症例 1）左卵巣腫瘍病理組織所見

類円形あるいは一部不整形の核を持ち，淡明な胞体を有する細胞が充実性に増殖していた．a：H＆E 染色 弱拡大，b：H＆E 染色 強拡大

Figure5　（症例 2）骨盤部造影ＣＴ検査

右骨盤内に 48mm 大の血流豊富な充実性腫瘍を認めた．

Figure6　（症例 2）右卵巣腫瘍肉眼写真

右卵巣腫瘍の割面は黄色調で出血を伴うスポンジ状であった．

Figure7　（症例 2）右卵巣腫瘍病理組織所見

類円形あるいは一部不整形の核を持ち，淡明から淡好酸性の胞体を有する腫瘍細胞が胞巣構造や線管構造を形成しながら充実性に増殖していた．また，腫瘍内は線維性血管間質によって区画されていた． a：H&E 染色 弱拡大，b：H&E 染色 強拡大

Figure1

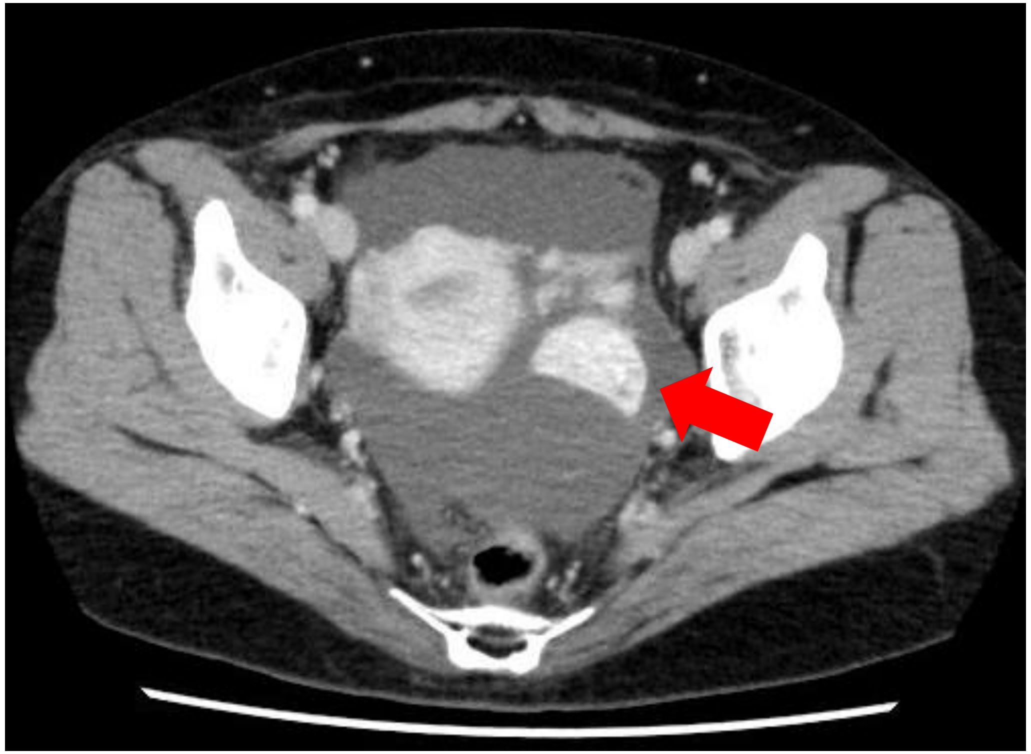

Figure2

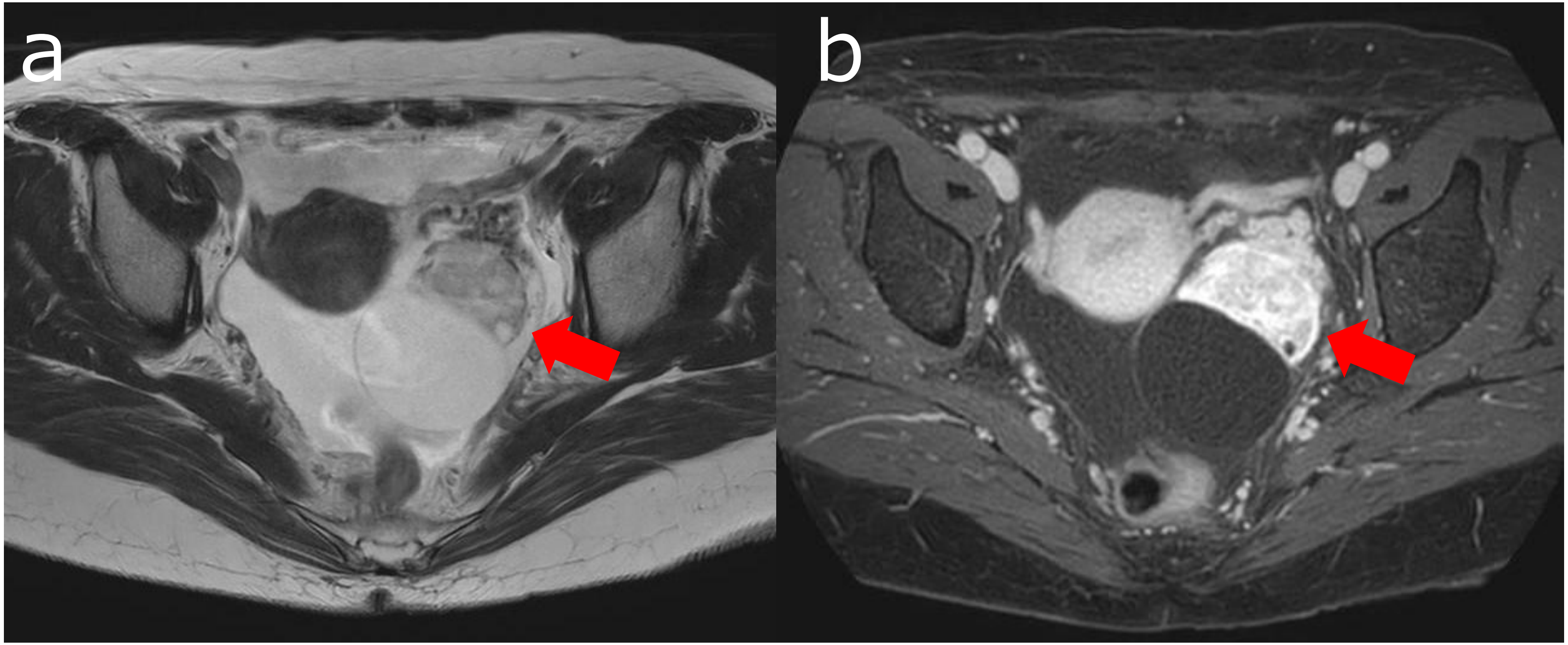

Figure3

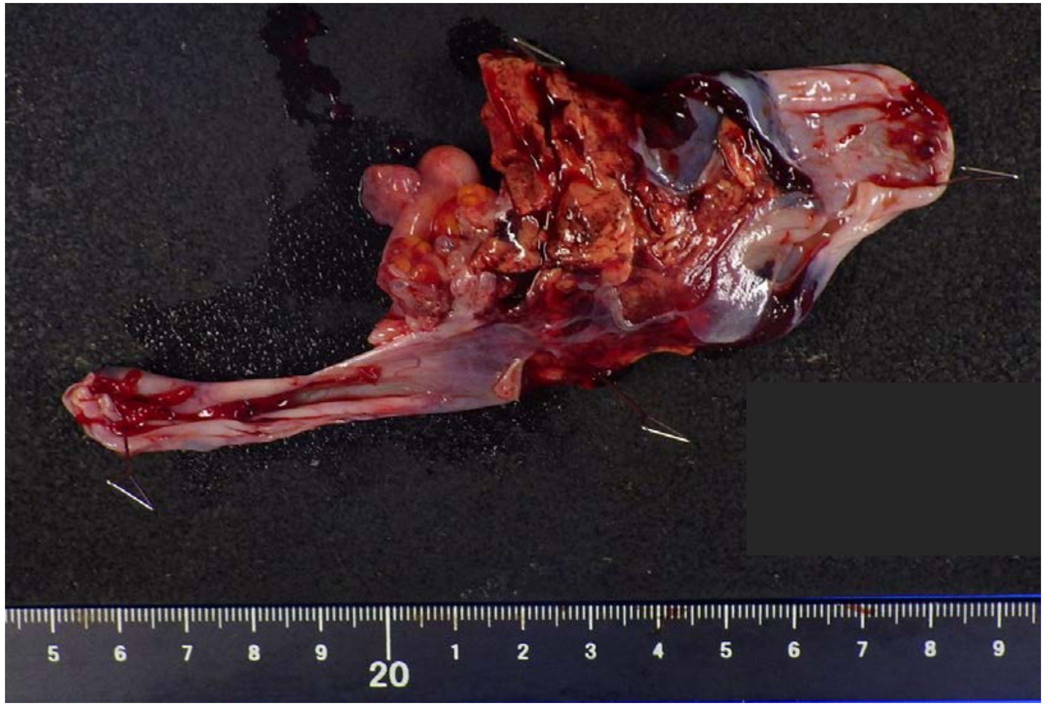

Figure4

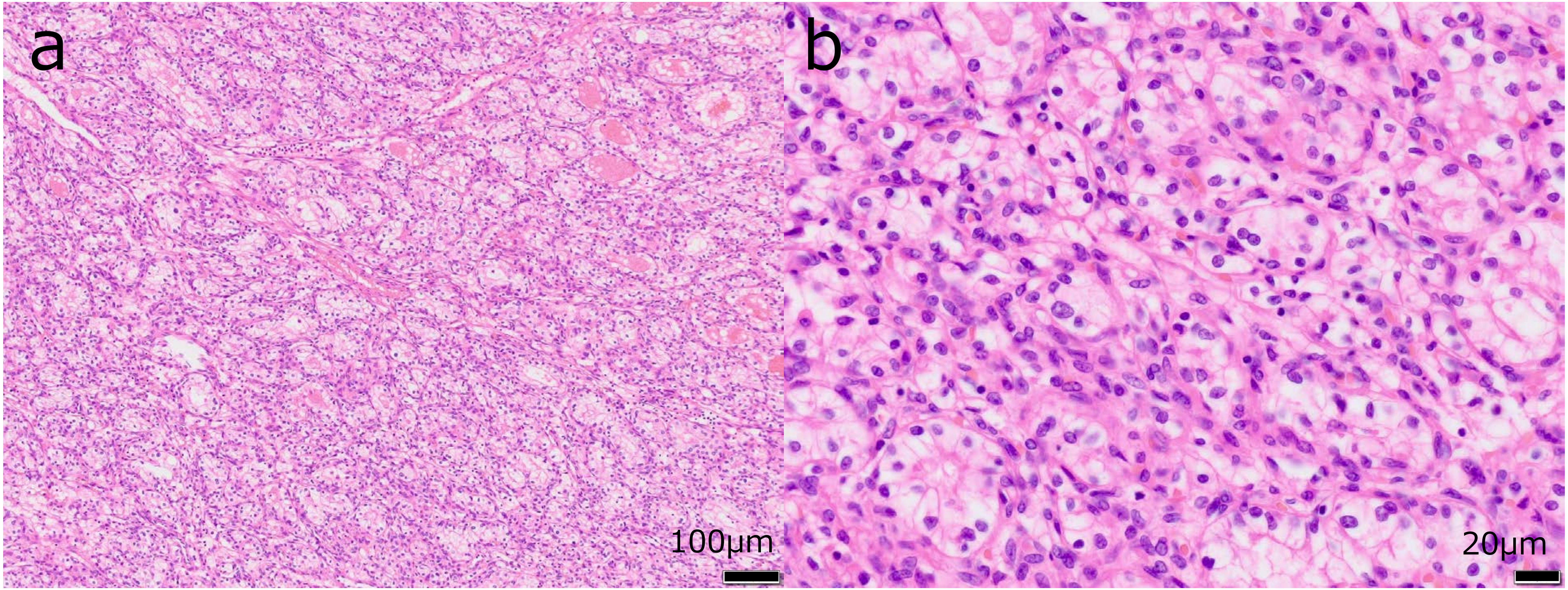

Figure5

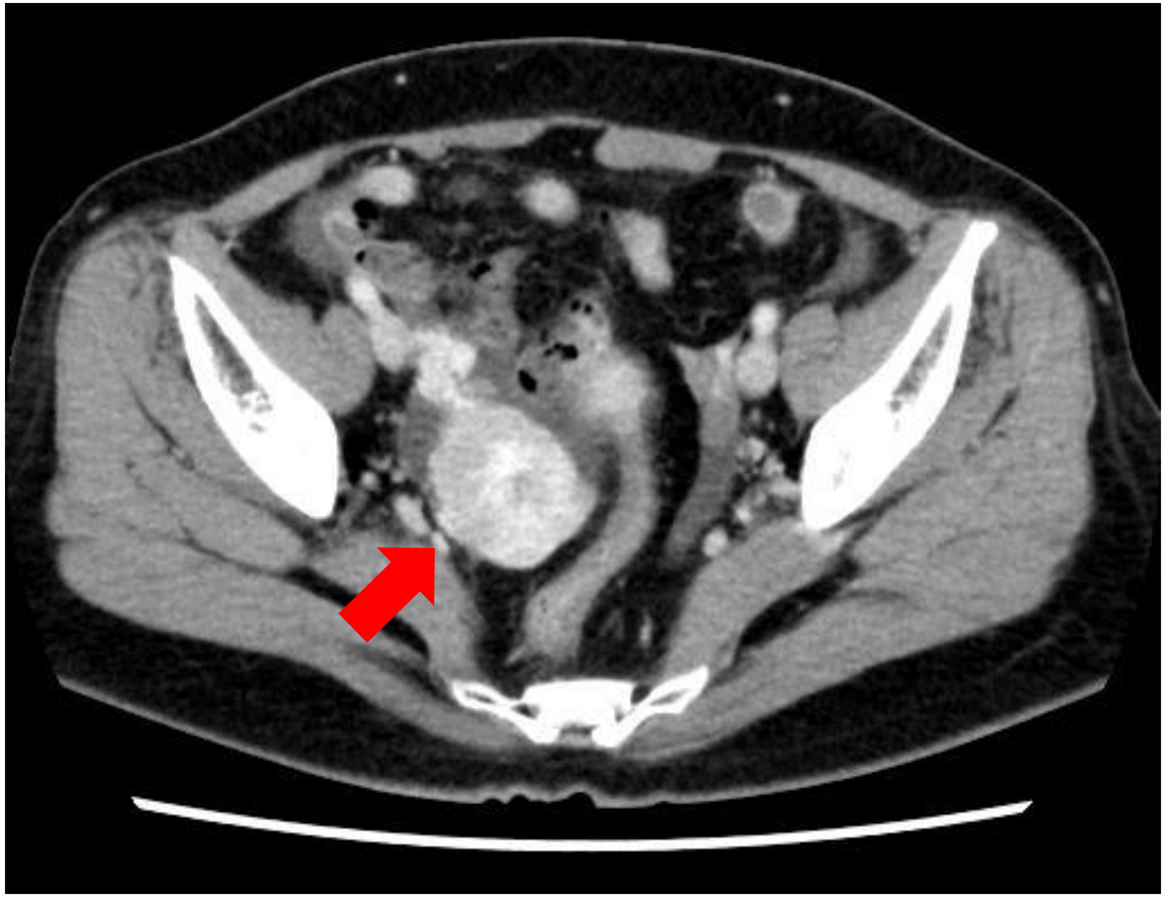

Figure6

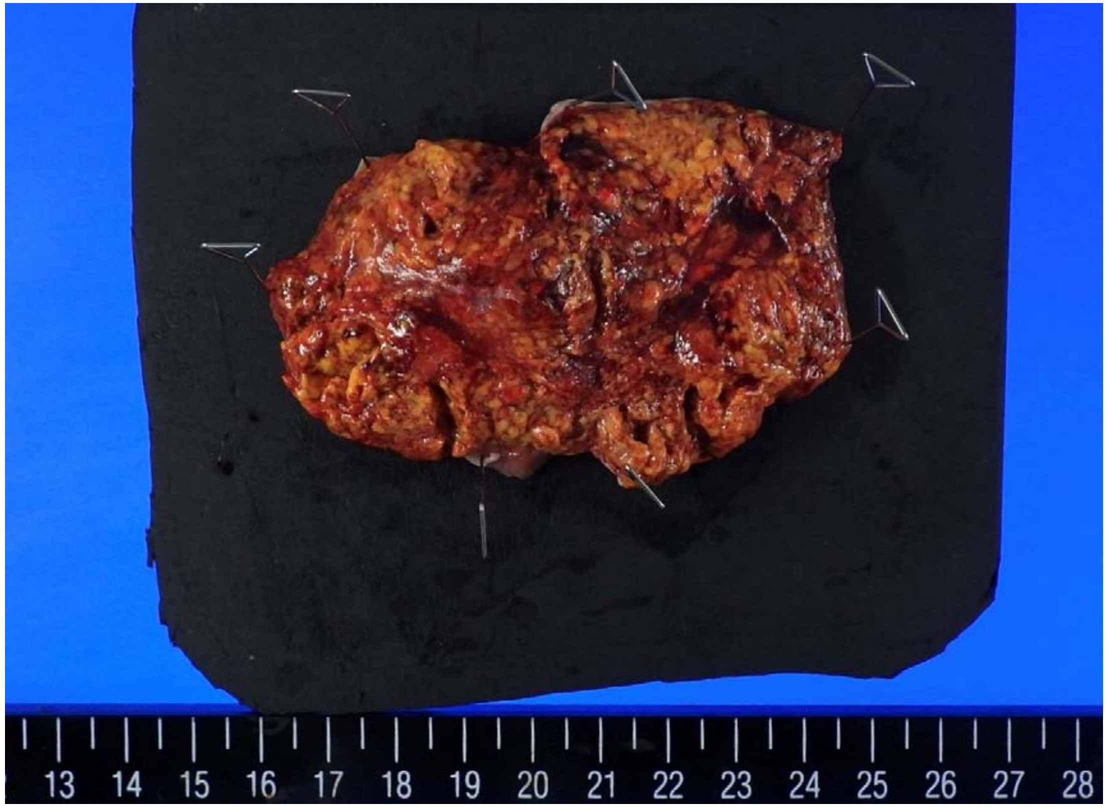

Figure7

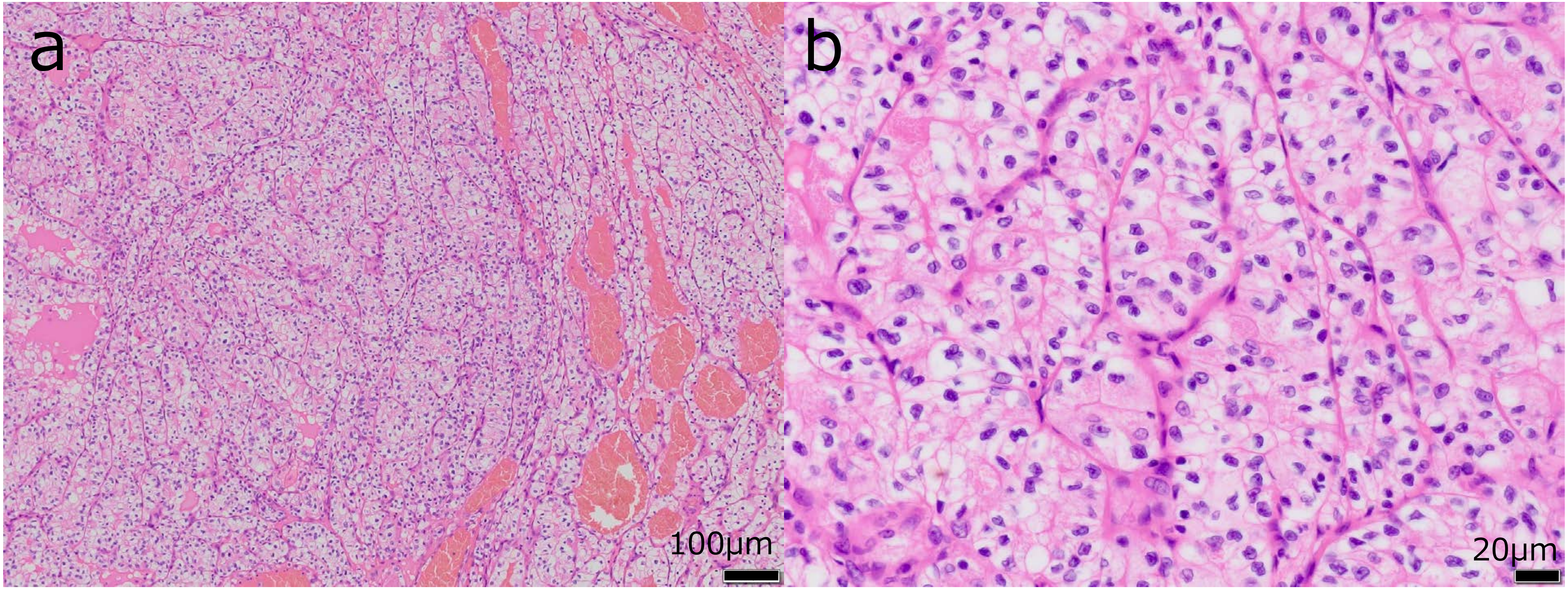

Table1. The summary of the cases.

References	Age (years)	Laterality of RCC	Laterality of Ovarian metastasis	First detected site	Time to metastases
Vorder 1957	64	Right	Bilateral	Kidney	11y
Stefani 1981	68	Right	Left	Kidney	3mo
Young 1992	48	Right	Left	Ovary	8mo
	62	Left	Right	Kidney	1y
	48	Left	Left	Synchronous	
Liu 1992	28	Right	Left	Kidney	7mo
Spencer 1993	40	Left	Bilateral	Ovary	7mo
Adachi 1994	46	Left	Bilateral	Kidney	3y
Fields 1996	54	Right	Left	Kidney	3y
Vara 1998	66	Right	Bilateral	Kidney	14y
Hammock 2003	48	Left	Right	Synchronous	
Insabato 2003	50	Right	Right	Kidney	1y
	49	Right	NA	Kidney	14mo
	17	Left	Left	Kidney	2y
Valappil 2004	61	Left	Bilateral	Kidney	7y
Kato 2006	52	Left	Right	Synchronous	
Stolnicu 2007	73	NA	Left	Kidney	NA
Toquero 2009	54	Left	Left	Synchronous	
Albrizio 2009	56	Right	Bilateral	Kidney	10y
Decoene 2011	47	Right	Left	Kidney	5y
Bauerova 2014	61	Right	Bilateral	Kidney	21y
Bohara 2015	48	Right	Right	Kidney	3y
Kostrzewa 2015	51	Left	Right	Kidney	4y
Liang 2016	60	Right	Right	Synchronous	
	NA	NA	Bilateral	Synchronous	
	48	Left	Right	Kidney	14mo
	37	Left	Bilateral	Kidney	8mo
	45	Right	Left	Kidney	30mo
	43	Right	Right	Kidney	20mo
	52	Left	Right	Kidney	10mo
	52	Right	Left	Ovary	NA
Uruc 2017	48	Right	Left	Kidney	22mo
Bhaskar 2017	45	Left	Left	Synchronous	
Porfyris 2018	82	Right	Left	Synchronous	
Karaosmanoglu 2019	52	Left	Left	Kidney	4y
Takayanagi 2019	66	Right	Right	Kidney	4y
Fujii 2021	58	Right	Bilateral	Kidney	8y
Snyder 2021	48	Left	Bilateral	Synchronous	
Younes 2023	56	Left	Left	Kidney	15y
Present case 2022	52	Left	Left	Kidney	9y
	56	Right	Right	Kidney	15y

Abbreviation: NA; not available, RCC; renal cell carcinoma, y; year, mo; month
